# Chitosan nano-formulation enhances stability and bactericidal activity of the lytic phage HK6

**DOI:** 10.1186/s12896-024-00934-6

**Published:** 2025-01-06

**Authors:** Hasnaa R. Temsaah, Karim Abdelkader, Amr E. Ahmed, Nada Elgiddawy, Zienab E. Eldin, Hend Ali Elshebrawy, Nahed Gomaa Kasem, Fatma A. El-Gohary, Ahmed. F. Azmy

**Affiliations:** 1 Biotechnology and Life Science Department, Faculty of Postgraduate Studies for Advanced Sciences (PSAS), Beni-Suef University, Beni-Suef, 62511 Egypt; 2 Department of Microbiology and Immunology, Faculty of Pharmacy, Beni-Suef University, Beni-Suef, 62511 Egypt; 3 Materials Science and Nanotechnology Department, Faculty of Postgraduate Studies for Advanced Sciences, Beni-Suef University, Beni-Suef, 62511 Egypt; 4 Department of Food Hygiene and Control, Faculty of Veterinary Medicine, Mansoura University, Mansoura, 35516 Egypt; 5 Department of Hygiene and Zoonoses, Faculty of Veterinary Medicine, Mansoura University, Mansoura, 35516 Egypt

**Keywords:** *Enterobacter cloacae*, Bacteriophage, Chitosan, Nanoparticles, Phage release

## Abstract

**Background:**

Successful treatment of pathogenic bacteria like *Enterobacter Cloacae* with bacteriophage (phage) counteract some hindrance such as phage stability and immunological clearance. Our research is focused on the encapsulation of phage HK6 within chitosan nanoparticles.

**Result:**

Encapsulation significantly improves stability, efficacy, and delivery of phages. Chitosan nanoparticles (CS-NPs) achieve a phage entrapment efficiency of 97%. Fourier-transform infrared spectroscopy (FT-IR) reveals shifts towards higher wavenumbers and a new peak, indicating amide bond formation and successful phage encapsulation. The average particle sizes for CS-NP and phage HK6 encapsulated CS-NPs were 180 ± 10 nm and 297 ± 18 nm, respectively. Scanning Electron Microscopy (SEM) and Transmission Electron Microscopy (TEM) analyses reveal that phage HK6 encapsulated CS-NPs are larger on average than CS-NPs, highlighting successful phage encapsulation. Encapsulated bacteriophages maintain its effectiveness at higher pH levels of 11 and 12. Both encapsulated and free bacteriophages are thermostable between 25 and 60 °C; while at higher temperatures (up to 80 °C), the encapsulated phage is thermally stable. Over four days, 70.57% of phages were released from encapsulated CS-NPs. Encapsulation of bacteriophage HK6 in CS-NPs enhances antibacterial activity within the first 2 h, compared to phage or nanoparticles alone.

**Conclusion:**

This suggests that the phage HK6 encapsulated CS-NPs exhibit potentiality as biocontrol agents against resistant microorganisms offering an alternative to phage alone.

**Graphical Abstract:**

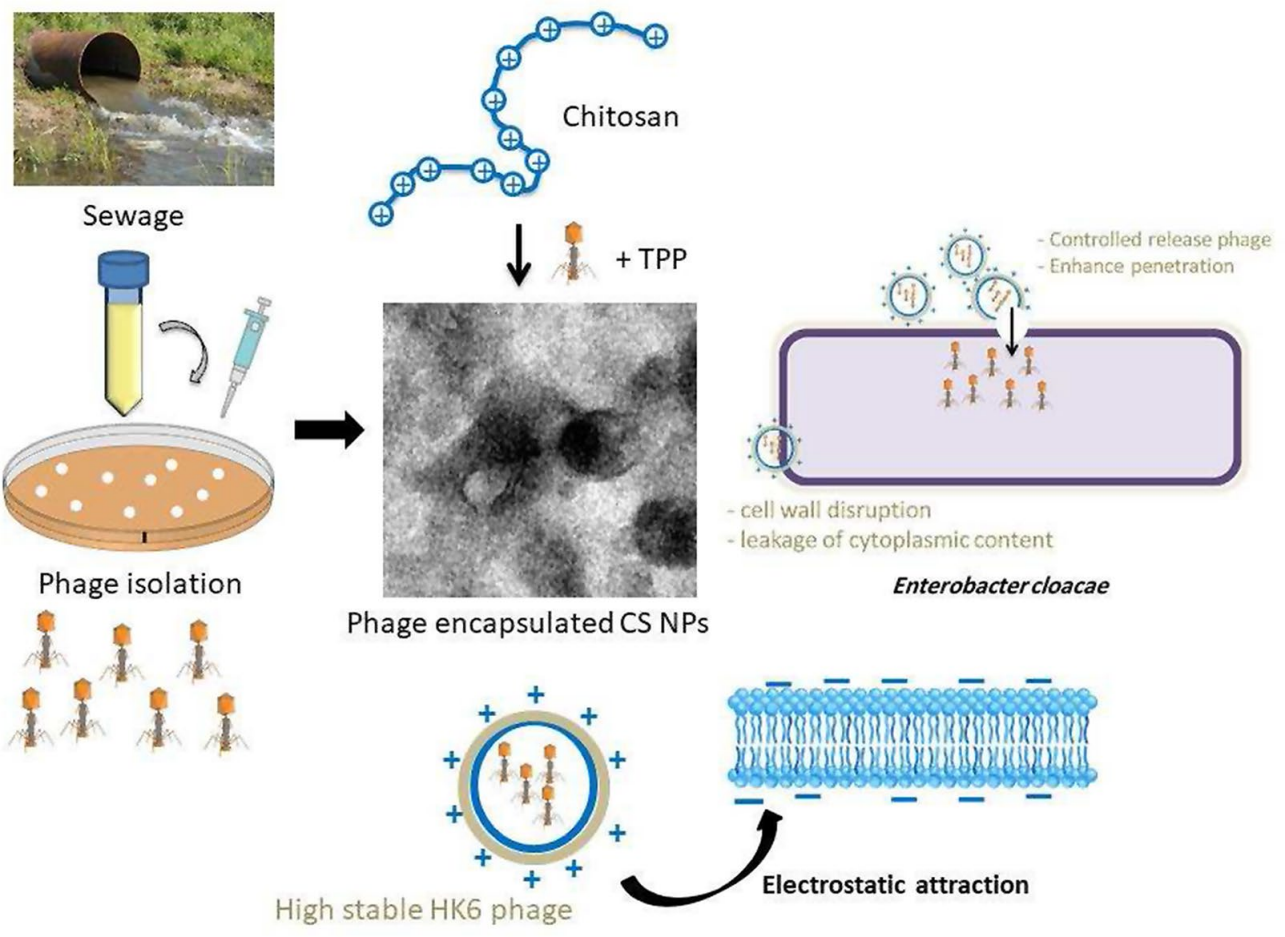

**Supplementary Information:**

The online version contains supplementary material available at 10.1186/s12896-024-00934-6.

## Introduction

*Enterobacter cloacae* (*E. cloacae)* is a Gram-negative bacterium that can be frequently detected in various sources such as food samples, water, plants, soil, a human body, and animals. *E. cloacae* is widely known to be the primary cause of nosocomial infections, including pneumonia, urinary tract infections, eye infections, bloodstream infections and those occurring in neonatal intensive care units, resulting in a considerable number of fatalities [1, 2]. *E. cloacae* is known as biofilm forming bacterium that infect catheters of hospital patients, leading to catheter-related infections [2]. Most species of *E. cloacae* have inherent resistance to many antibiotics, including first-generation cephalosporins, ampicillin, amoxicillin, and amoxicillin-clavulanate [1]. Recently, many studies that focus in controlling the antimicrobial resistance, have been suggested phages as a valuable alternative to antibiotics [3].

Phages, which are the most numerous biological entities on earth, exert a major effect over the microbial community’s ecological equilibrium. They are viruses that specifically target and destroy bacteria [4]. The majority of phages consist of a protein-based tail that aids in precise recognition of a receptor located on the surface of the host bacterium and a head that encapsulates the genome [5]. Phages, in contrast to antibiotics, proliferate at the site of infection, enabling their rapid spread without dosage restrictions; furthermore, they offer cost-effectiveness [6].

Despite the considerable bacteriolytic capabilities of phages against numerous species, their application in clinical environments is still limited. This is due to the stability of phage preparations, target-site-specific delivery, the antibody-mediated inactivation of phages and clearance by the reticuloendothelial system of the recipient, as well as inadequate phage titers employed during treatment that can lead to the development of bacterial resistance [7]. Particularly, phage environmental stability (thermal, pH and storage stability) is crucial when industrial or biocontrol applications are intended. Bacteriophages respond variably to the external conditions, making it challenging to select the right phage candidates for diverse applications. For instance, tailed phages are found to be more resilient to harsh conditions than tailless counterparts. In addition, phages optimally infect and replicate within their bacterial host in pH range of 6–9. Outside this range, their infectivity and stability are significantly reduced that may limit their broad application. Therefore, exploring strategies for improving phage stability is needed [8]. The encapsulation of phages within nanoparticles represents effective strategies that include phage protection from the external environment, an efficient active delivery in addition to sustained release effect [9].

Chitosan, obtained by N-deacetylation of chitin, is a naturally occurring polycationic polysaccharide that has found diverse applications as carriers in medicine, agriculture, and food packaging. This is primarily attributed to its favorable characteristics, such as biodegradability, biocompatibility, low immunogenicity, along with its high charge density, mucoadhesiveness, and lack of toxicity [10]. Chitosan nanoparticles (CS-NPs) amplified their effectiveness due to decrease in particle size. Numerous studies have revealed CS-NPs as carriers for large biological molecules, such as peptides, proteins, nucleic acids, and plasmids. They enhance anticancer, antioxidant, antimicrobial, and anti-inflammatory properties in comparison to chitosan alone [11]. Recent research has shown increased emphasis on elucidating the potential of synergistic interactions between natural polymers and phage. Consequently, the utilization of phage-natural-nano therapy, for biological purposes, constitutes a noteworthy advancement in the field of nanoscience [12].

Previously, we have isolated and characterized the Enterobacter phage HK6 (accession no. PP337149) against the multidrug-resistant *E. cloacae* EC21 food isolate [13]. The phage showed acceptable kinetic, bacteriolytic and stability features, making it a promising candidate for food application. In the current study, we have investigated whether its encapsulation in CS-NPs could push the stability and antibacterial activity boundaries towards a better performance, making it suitable for broader application.

## Materials and methods

### Materials and bacterial strains

Chitosan (MW, 75 kDa), sodium tripolyphosphate (TPP), acetic acid, dimethyl sulfoxide (DMSO), and ethanol were purchased from Sigma (Sigma-Aldrich Co, St. Louis, Missouri, USA). terephthalic acid, benzoic acid, NaOH, HCl and Dimethyl Formamide (DMF) (Chem-Lab, Belgium). LB broth, SM Buffer is a mixture of 100mM Sodium chloride, 8mM Magnesium sulphate and 0.01% gelatin.

Twelve previously characterized food- recovered *E. cloacae* strains [13] were included in the current study to evaluate the antibacterial activity of the free and encapsulated Enterobacter phage HK6. Identification and antibiotic sensitivity of the of the included strains were listed in Table 1.

**Table 1:** Antibacterial activity of phage, chitosan and phage encapsulated CS-NPs against different strains of *Enterobacter cloacae*

Enterobacter cloacae Strains	HK6	Chitosan only	Phage encapsulated in CS-NPs	Antibiotic resistance [13]
EC3	**+**	**-**	**+**	AMP, AMC, CAZ, CXT
EC 4	**-**	**-**	**-**	AMP, AMC. CXT
EC 7	**+**	**+**	**++**	AMP, AMC, CAZ, CXT
EC 10	**-**	**+**	**+**	AMP, AMC. CXT
EC 11	**+**	**+**	**++**	AMP, AMC, CAZ, CXT
EC 12	**-**	**-**	**-**	AMP, AMC. CXT
EC 13	**-**	**+**	**+**	AMP, AMC. CXT
EC 21	**+**	**+**	**++**	AMP, AMC. CXT
EC 25	**+**	**+**	**+**	AMP, AMC, CAZ, CXT, CIP, SXT
EC 26	**-**	**-**	**-**	AMP, AMC. CXT
EC 29	**+**	**-**	**+**	AMP, AMC, CAZ, CXT, CIP
EC 34	**+**	**+**	**++**	AMP, AMC, CAZ, CXT

(-) no zone of inhibition

(+) zone of inhibition with diameter from 5–7 mm

(++) zone of inhibition with diameter from 7–11 mm

AMP Ampicillin, AMC Amoxycillin – clavulanic, CAZ Ceftazidime, CXT Ceftriaxone, CIP Ciprofloxacin, SXT Sulfamethoxazole-trimethoprim

### Bacteriophage isolation and purification

To isolate bacteriophage, samples were collected from different sewage outlets, then centrifuged and filtered using syringe filter (0.45-µm). Afterwards, the filtered samples were mixed with LB and enriched with *E. cloacae* strains. The mixtures were incubated for 18–20 h at 37 ºC with shaking. spot on-lawn [14] and double layer assays [15] were applied on the collected supernatant individually to determine potential bacteriophage lytic activity Purification of bacteriophage HK6 was performed by repeated plating of each individual plaque up to seven times.

The isolated phage was characterized and sequenced (sequence of isolated phage was submitted under GenBank accession number HK6 PP337149).

The titer of the phage was determined by serial dilution of stock of isolated phage in SM buffer using the Double Layer Assay (DLA). In summary, *E. cloacae* culture was diluted in 0.7% LB soft agar and thoroughly mixed prior to pouring onto plain LB agar plates. Serial 10-fold dilutions of the phage suspension were prepared, and 10 µL of each dilution was spotted onto an inoculated plate. The plates were incubated overnight at 37 °C, and the plaques on each plate were counted.

### Preparation of phage HK6 encapsulated CS-NPs

CS-NPs were synthesized through the dissolution of 0.3 g chitosan in 1% acetic acid with continuous stirring with a magnetic stir for 1 h. The mixture was vortexed and sonicated for 5 and 30 min, respectively. The pH of the solution was adjusted to 5.5 through the addition of 0.1 M sodium hydroxide with gentle stirring. The phage suspension (10^7^ PFU/mL) was added dropwise in chitosan solution with gentle agitation. Phage adsorbed chitosan was encapsulated using a dropwise addition of 0.3% of TPP. With Proper sonication for 20 min. The solution was stored at 4 °C until used [16]. Free CS-NPs were prepared using the same methodology without adding phage solution.

### The encapsulation efficiency of CS-NPs

To determine Entrapment efficiency (EE %), phage HK6 encapsulated CS-NPs were centrifuged at 19,980 × g for 30 min. The quantity of unentrapped phage HK6 in the supernatant solution (free phage) was then calculated using the following equation through DLA assay [17].

$${\rm{EE\% = }}{\matrix{{\rm{Total}}\,{\rm{amount}}\,{\rm{of}}\,{\rm{phage - }} \hfill \cr {\rm{ amount}}\,{\rm{of}}\,{\rm{unentrapped}}\,{\rm{phage}} \hfill \cr} \over {{\rm{Total}}\,{\rm{amount}}\,{\rm{of}}\,{\rm{phage}}}}{\rm{ \times 100}}$$

### Characterization of chitosan Nano particle and phage HK6 encapsulated CS-NPs Methods

Fourier transform infrared (FT-IR) spectra were recorded by the KBr pellet technique on a Bruker (Vertex 70 FT-IR) spectrometer in the range from 4000 to 400 cm − 1. The average size of CS-NPs was measured in 18 megohm-cm deionized water by dynamic light scattering (DLS). Zeta-potential was measured on a Zeta sizer nano-ZS 90 (Malvern Instruments, UK) at 25 °C in clear disposable zeta cells.

The morphology of the synthesized CS-NPs was recorded using a Field emission scanning electron microscope (FE-SEM) with JEOL JEM-2100 and an internal charge coupled device (CCD) camera.

Transmission electron microscope (TEM) images were taken by JEOL-JEM 2100 (Japan) with an acceleration voltage of 200 kV.

### Antibacterial activity of phage HK6, chitosan and phage HK6 encapsulated CS-NPs

Antibacterial activity of isolated phage HK6 was tested against different strains of *E. cloacae* whish isolated and characterized biochemically using the automated VITEK. Firstly, overnight bacterial cultured was spread-plated on Muller Hinton agar plate. 5 µL of phage HK6, CS-NPs and phage HK6 encapsulated nanoparticles were spotted separately on seeded plates. The plates were allowed to dry and incubated at 37 ◦C for 24 h. then the inhibition zones were measured to determine the antimicrobial potency of each formulation [18].

### Determination of Thermal and pH Stability of phage HK6 and phage HK6 encapsulated CS-NPs

For pH stability, phage sample and phage encapsulated CS-NPs were incubated in different values of pH buffer 3, 5, 7, 9, 10, 11 and 12 (Britton Robinson buffer) for 1 h. at 4 ◦C. after incubation, phage samples were removed, serially diluted, and assayed using the DLA method to determine the titer of the surviving phages [19].

For thermal stability assay, 1 mL of previously mentioned phage preparations in SM buffer was incubated at 25^o^C, 50^o^C, 60^o^C, 70^o^C and 80^o^C for 1 h. After incubation, the phage titer was calculated as described above using the DLA method [20].

The experiments were repeated in triplicate and statistically analyzed for the significance.

### Time-killing curve

*E. cloacae* cells were grown overnight in LB at 37 °C and regrown in fresh medium for 2 to 3 h. To achieve a final density of 10^5^ CFU/mL (OD_600_ = 0.5). Bacterial suspension was centrifuged and the cells were suspended in SM buffer to a conc of 10^5^ CFU/mL. 100 µl of cell suspension was incubated with an equal amount of SM buffer (as positive control), phage HK6, CS-NPs, and phage HK6 encapsulated CS-NPs. These mixtures were incubated at 37 ^o^C for 24 h. the Viable bacterial cells were counted in each mixture at different time interval (0, 4, 8, 12, and 24 h) using the viable count technique [21]. All trials were conducted in triplicate and statistically analyzed for the significance.

### In vitro phage HK6 release assay

Phage release from phage encapsulated CS-NPs was detected by Biciconic Acid (BCA) assay to determine the protein concentration [1]. 1 mL of phage HK6 encapsulated CS-NPs was transferred into a tube containing 5 mL of Britton-Robinson buffer, which was maintained at 37 °C and a pH of 7. At predetermined time intervals (0, 1, 2, 3, 4, 6, 12, 24, 48 and 96 h), 1 mL of the sample was removed after centrifugation at 11,000 × g for 30 min, and then replaced with 1 mL fresh Britton Robinson buffer. The sample from each time interval was analyzed by BCA assay [22]. The **r**elease assay was performed in triplicate for the significance.

### Evaluation of Cytotoxicity using MTT Assay

Epithelial cells (hTERT RPE-1) were cultured in DMEM-F12 medium supplemented with 1% antibiotic-antimycotic mixture (10,000U/ml Potassium Penicillin, 10,000 µg/ml Streptomycin Sulfate and 25 µg/ml Amphotericin B) and 1% L-glutamine in 96-well microtiter plastic plates at 37 ^o^ C for 24 h under 5% CO2. Media was aspirated, fresh medium (without serum) was added, and cells were incubated either alone (negative control), chitosan nanoparticles alone (0.01–1 mg/ml) or with different concentrations of phage HK6 encapsulated CS-NPs (10^12^, 10^11^, or 10^10^ PFU/mL) (10000 and 15000 cells/well) for 48 h at 37 °C before toxicity evaluation by the MTT assay. The absorbance was evaluated at a wavelength of 595 nm with a reference wavelength of 620 nm [23].

#### Statistical analysis

The data were analyzed using SPSS 20 software using T–test. A p-value less than 0.05 was considered statistically significant For MTT assay, A statistical significance was tested between samples and negative control (cells with vehicle) using independent t-test by SPSS 11 program.

## Results

### Bacteriophage preparation and Encapsulation Efficiency

The final titer of phage HK6 encapsulated CS-NPs was 10^7^ plaque forming units per milliliter (PFU/mL). We determined the entrapment efficiency from the amount of unentrapped phage which was found to be (3 × 10 ^5^ PFU/mL) this indicates the capsulation efficiency of about 97%, of total phage HK6 was encapsulated in the CS-NPs.

### Characterization of chitosan and phage HK6 encapsulated CS-NPs

#### FTIR analysis

**Fig. 1:**
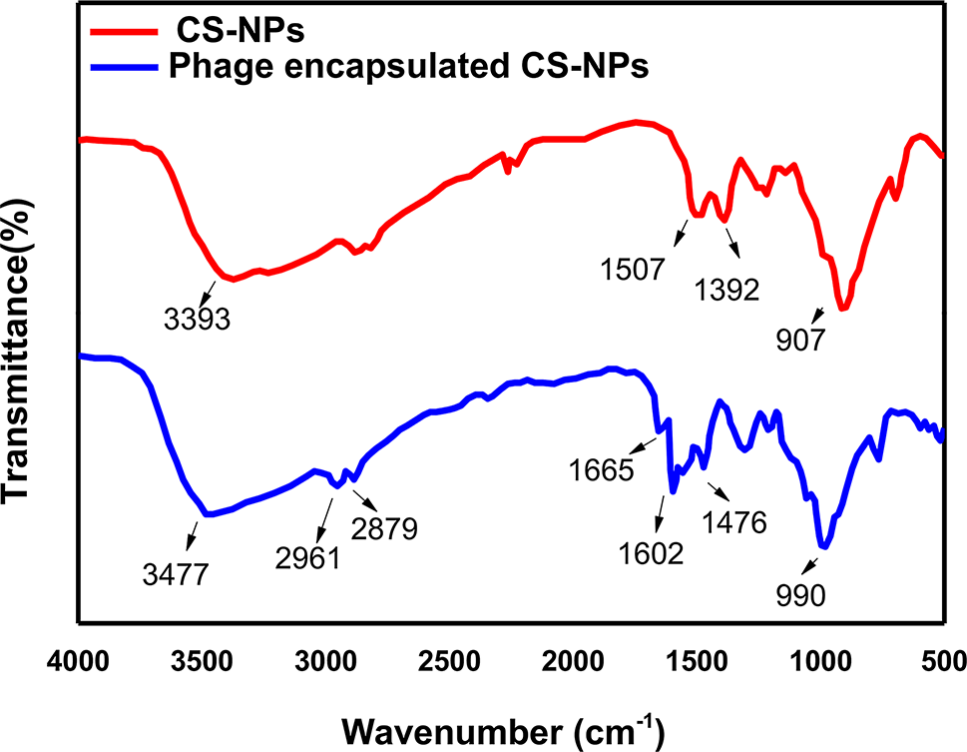
FTIR spectra of CS-NPs () showed a broad band at 3393 cm − 1 corresponds to intermolecular hydrogen bond stretching vibrations of (O–H and N–H) groups. The peaks at 1507 cm − 1 is attributed to CH bending and 1392 cm^− 1^ are attributed to the CH2 stretching vibration; and peak at 907 cm^− 1^ characteristic to P = O stretching vibration. FTIR of phage encapsulated CS-NPs (), the peak at 3393 cm − 1 (–NH2 groups stretching vibration) was shifted to 3477 cm^− 1^ attributing to the occurrence of phage encapsulated. In addition, the peaks at 1507 and 1392 cm^− 1^ in CS-NPs were shifted to 1602 and 1476 cm − 1 in CS-NPs, with the presence of a peak at 1665 cm^-1 is typically associated with the C = O (carbonyl) stretching vibration

The FTIR spectrum of CS-NPs and phage HK6 encapsulated CS-NPs were showed in Fig. 1. The CS-NPs showed a broad band at 3393 cm − 1 corresponds to intermolecular hydrogen bond stretching vibrations of (O–H and N–H) groups. The peaks at 1507 cm^− 1^ is attributed to CH bending and 1392 cm^− 1^ is attributed to the CH2 stretching vibration; and peak at 907 cm − 1 characteristic to P = O stretching vibration.

Compared with phage HK6 encapsulated CS-NPs, the peak at 3393 cm^− 1^ (–NH2 groups stretching vibration) was shifted to 3477 cm^− 1^ attributing to the occurrence of phage encapsulated. In addition, the peaks at 1507 and 1392 cm − 1 in CS-NPs were shifted to 1602 and 1476 cm − 1 in CS-NPs, with the presence of a peak at 1665 cm^− 1^ is typically associated with the C = O (carbonyl) stretching vibration.

#### DLS and zeta potential measurements

**Fig. 2:**
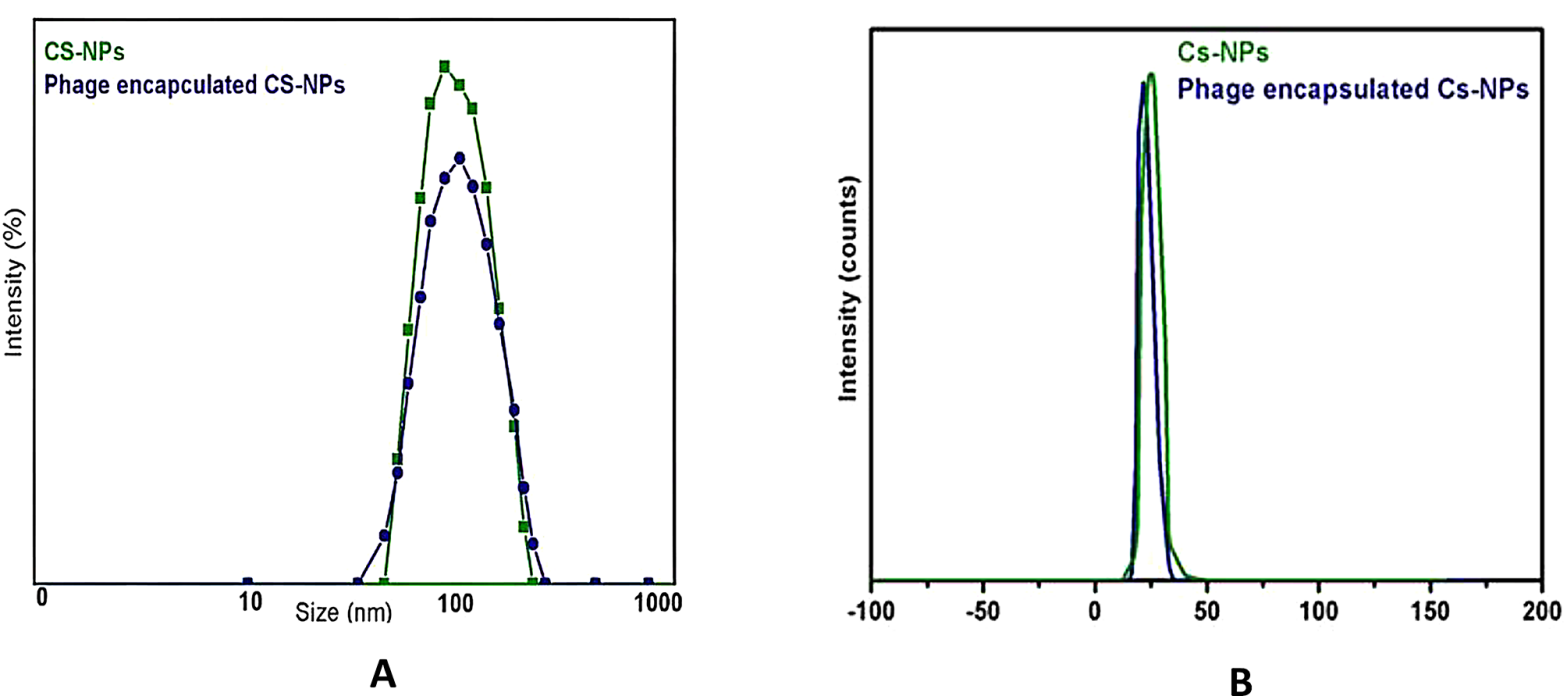
(**A**) Size distribution of CS-NPs () and phage encapsulated CS-NPs () show was found to be 180 ± 10 nm and 297 ± 18 nm, respectively, with a narrow size distribution (PDI values were 0.3 to 0.4). (**B**) Zeta potential of CS-NPs () and phage encapsulated CS-NPs () was + 33 ± 6 mV and + 34 ± 3 mV, respectively

As showed in Fig. 2, the diameter size of CS-NPs and phage HK6 encapsulated CS-NPs were found to be 180 ± 10 nm and 297 ± 18 nm, respectively, with a narrow size distribution (PDI values were 0.3 to 0.4). The zeta potential of CS-NPs and phage HK6 encapsulated CS-NPs was + 33 ± 6 mV and + 34 ± 3 mV, respectively.

#### Morphological characterization

**Fig. 3:**
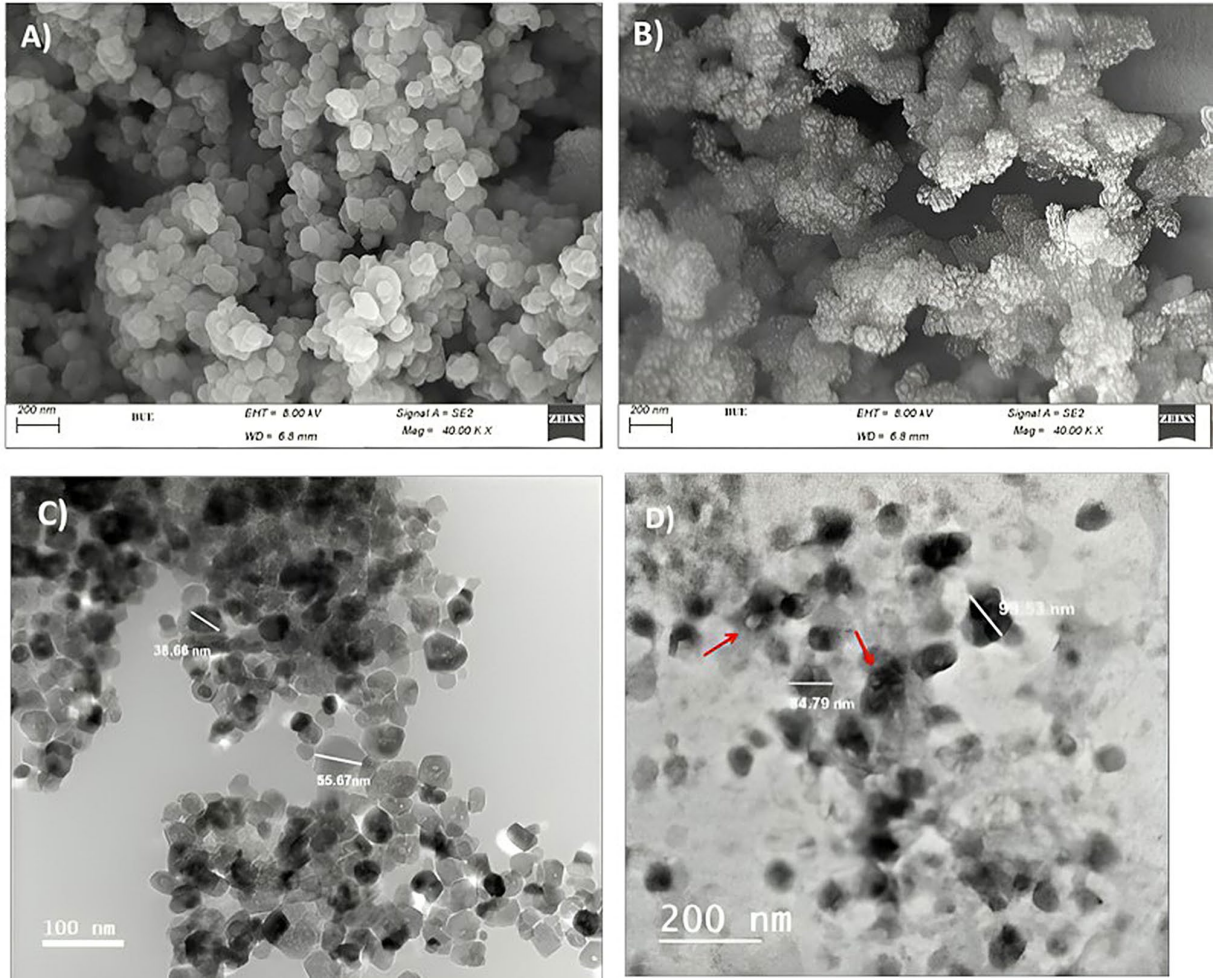
SEM images of CS-NPs (**A**) and phage encapsulated CS-NPs (**B**). TEM images of CS-NPs (**C**) and phage encapsulated CS-NPs (**D**)

The morphology of CS-NPs and Phage HK6 encapsulated CS-NPs were also investigated by SEM and TEM imaging. As shown in SEM images (Fig. 3), CS-NPs appear as spherical particles with a uniform shape which, affirm the formation of a stable NP dispersion.

TEM analysis was evident for the formulation of the nanoparticles in the solution and show particles of chitosan in the range of nanoscale (average 55.26 nm) with increasing the average size of CS-NPs (99.53 nm) indicating the phage encapsulated CS-NPs (Fig. 3).

### Antibacterial activity of phage HK6, chitosan and phage HK6 encapsulated CS-NPs

The evaluation of antibacterial efficacy across a range of *E. cloacae* bacterial strains revealed that phage HK6 encapsulated CS-NPs significantly enhanced antibacterial activity in comparison to the application of either phage or chitosan alone (Table 1). This finding underscores the synergistic potential inherent in the integration of phage and chitosan within nanoparticulate delivery systems.

### Temperature and pH stability

**Fig. 4:**
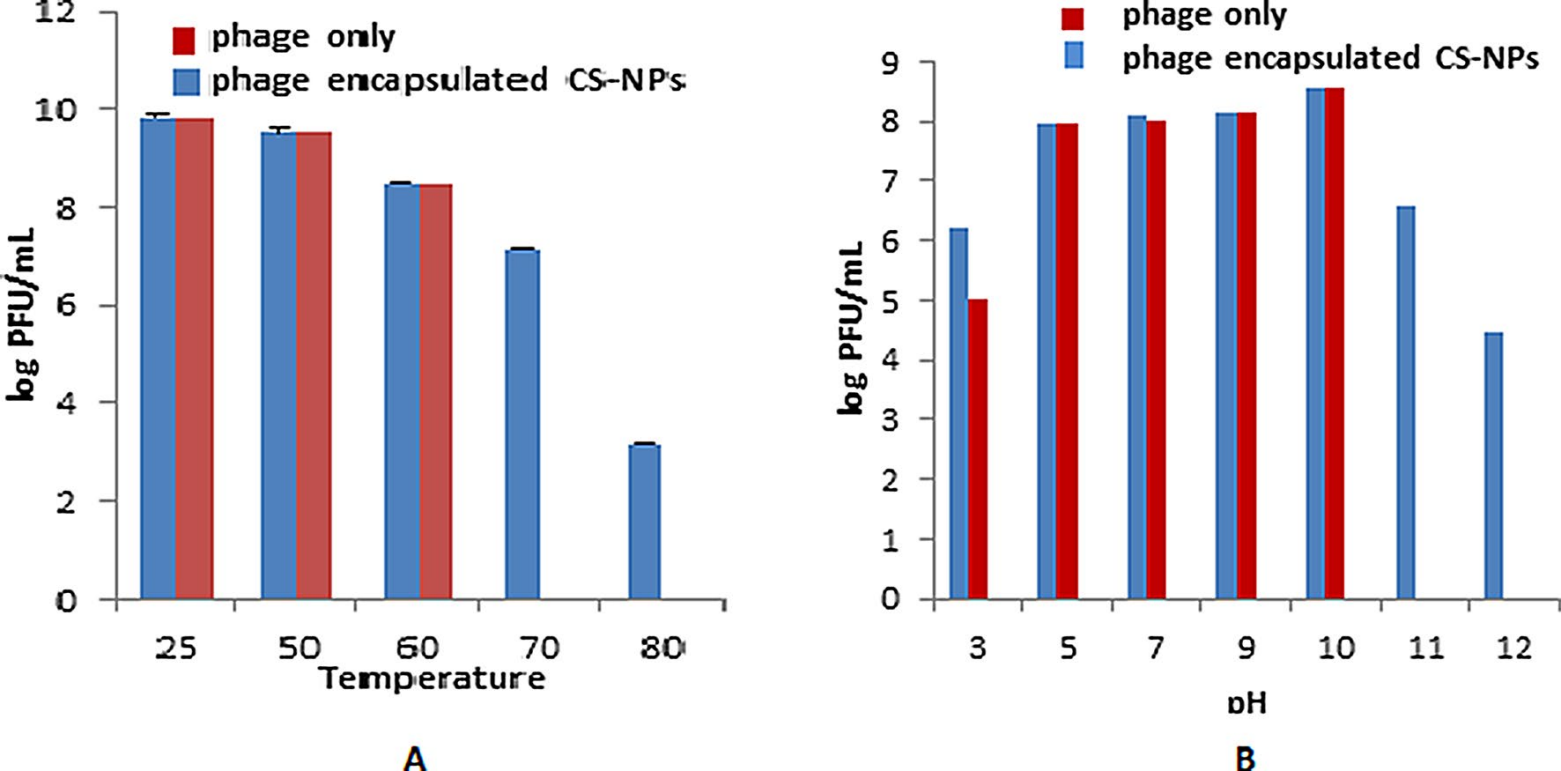
Thermal and pH stability test of phage and phage encapsulated CS-NPs. (**A**) Thermal tolerance, and (**B**) pH stability of free phage  and phage encapsulated CS-NPs , respectively Thermal tolerance was performed at different temperatures (25, 50, 60,70 and 80) for an hour and titer of surviving bacteriophages was calculated. pH tolerance was performed for 60 min at different pH (5–12) values at 4 °C. Data showed the log of the phage forming unit. Data shown are the mean of three replicates and error bars show the deviation in the values

To evaluate the thermal stability of free and encapsulated phage HK6, the set of experiments was performed in the spectrum of temperatures. According to the results, at 25 °C, 50 °C and 60 °C, both the free and encapsulated phage HK6 had the same level of activity, suggesting no notable difference in their heat tolerance with different temperature range. However, at elevated temperatures of 70 °C and 80 °C, the encapsulated phage HK6 demonstrated a slight thermal stability compared to their free state (Fig. 4).

The free and encapsulated phage HK6 activity and stability were measured under different pH (pH 3, 5, 7, 9, 10, 11, and 12). It has been observed that both forms of the phage can tolerate and inhibit the growth of bacteria within this pH range. The pH that showed the highest phage activity was at pH 10 for both free and encapsulated phage HK6. However, the encapsulated phage exhibited sustained effectiveness at elevated pH levels (pH 11 and pH 12), indicating enhanced resilience of the encapsulated phage in alkaline conditions (Fig. 4).

### Time killing assay

**Fig. 5:**
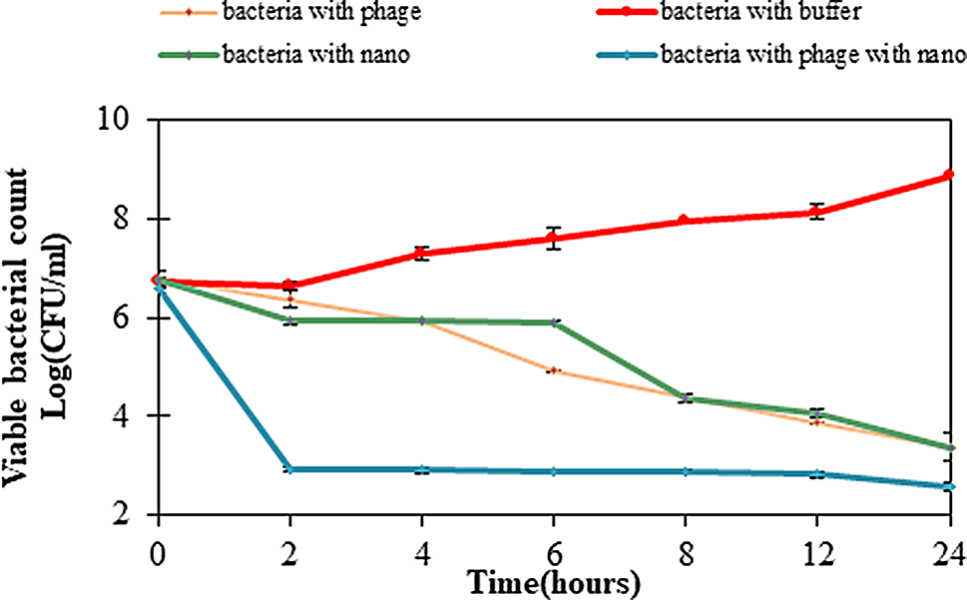
Time-killing curves for E. cloacae by phage only (), CS-NPs () only and phage encapsulated CS-NPs () and Non- treated bacterial cultures as a positive control (), viable cells were counted over 24 h. Show that bacteriophage encapsulated CS-NPs exhibited significant and rapid antibacterial activity within 2 h post-exposure. Activity was markedly superior compared to that of bacteriophage or CS-NPs alone

The antibacterial efficacy of phage HK6 encapsulated within CS-NPs was evaluated using a time-kill assay at a concentration of 10^7^ PFU/mL. The results indicated that phage HK6 encapsulated CS-NPs exhibited significant and rapid antibacterial activity within 2 h of exposure. activity was markedly superior compared to that of phage or CS-NPs alone (Fig. 5).

### Release study

**Fig. 6:**
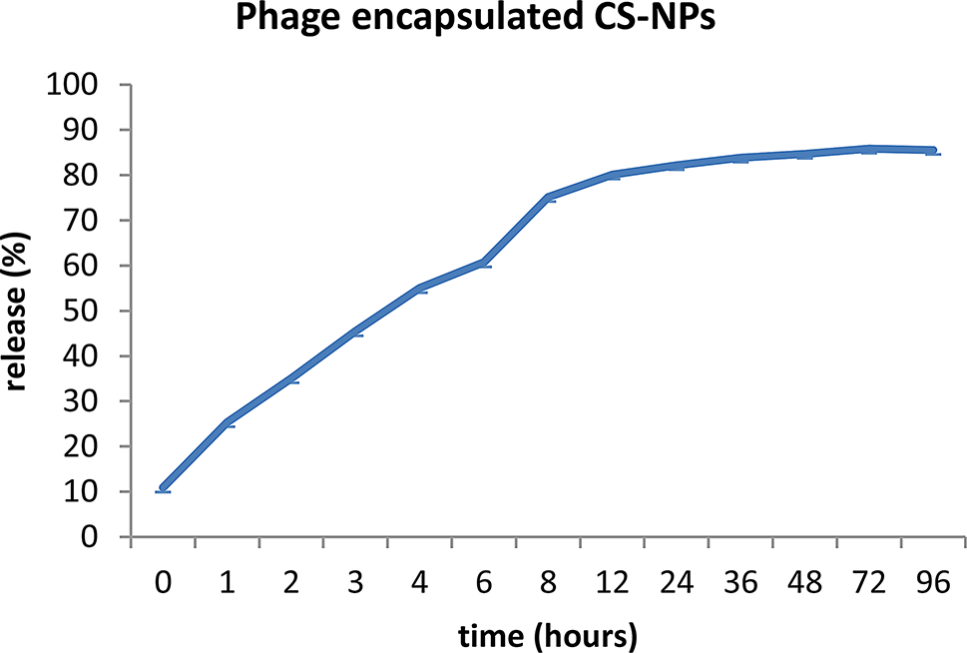
The release rate of the phage from phage encapsulated CS-NPs was tested by calculating the titer of the phage at different time points

The quantification of the in vitro release kinetics of phage HK6 encapsulated CS-NPs revealed that approximately 50% of the phage HK6 encapsulated within the CS-NPs were liberated into the buffer within the first 6 h then with approximately controlled release over a period of four days (Fig. 6).

### Cytotoxicity by MTT Assay

**Fig. 7:**
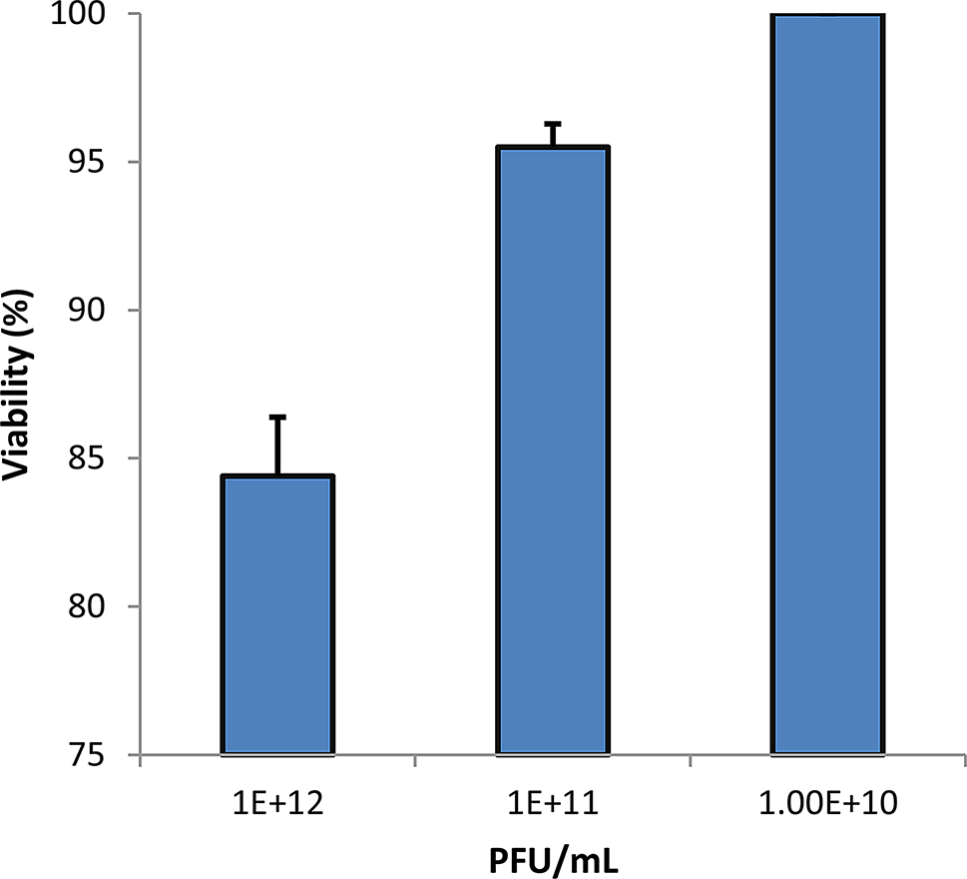
Cell viability % of epithelial cell line assessed by MTT assay by treatment with varying concentration (10^12^,10^11^ and 10^10^ PFU/mL) of phage encapsulated CS-NPs

The cytotoxic effects of varying concentrations of phage HK6 encapsulated in CS-NPs were quantitatively assessed using the MTT assay across a range of titers (10^12^, 10^11^, or 10^10^ PFU/mL) seeded on cell cultures. The investigation revealed that up to a titer of 10^10^ PFU/mL, the cell viability was 100%. However, an increase in titer to 10^11^ PFU/mL and 10^12^ PFU/mL was associated with a dose-dependent viability reduction to 95% and 85% respectively (Fig. 7). Parallel experiment using chitosan nanoparticle alone (0.01–1 mg/mL) showed neglectable change in cell viability (Figure S1 in the supplementary material).

## Discussion

In recent years, the majority of bacteria have acquired the ability to develop resistance to various classes of antibiotics; consequently, antibiotic resistance is among the greatest threats to global health [18].

An emerging alternative strategy for managing this infection involves the use of bacteriophages, which are viruses designed to infect and kill bacteria exclusively. Notably, these viruses lack any risk to humans, animals, or plants [6, 24]. Although phage therapy is effective but some drawbacks; like inadequate phage titers, degradation and inactivation by acids and gastric enzymes [25, 26] can give rise to bacterial resistance and treatment failure [7].

In this study, a phage HK6 encapsulated CS-NPs for biological control of *E. cloacae* infection was synthesized and characterized. The selection of chitosan for encapsulating phage is based on its biocompatibility, biodegradability, nontoxicity, and antimicrobial activity. its positive charge aids in the encapsulation of negatively charged molecules such as phages [27]. Chitosan exerts its antibacterial effect through its binding affinity for negatively charged bacterial cell walls, resulting in cellular disruption and alteration of membrane permeability [17].

The encapsulation efficiency (EE %) of phage HK6 in CS-NPs was 97%. This percentage accounts for maximal phage delivery to the target site during the treatment of bacterial infections.

Our study confirms that the phage HK6 encapsulated CS-NPs, this combination not only enhances the bioavailability and stability of the phage but also utilizes the inherent antimicrobial properties of chitosan, presenting a synergistic approach to effectively combat bacterial pathogens that surpasses the effectiveness of each individual component alone [17, 28].

On the basis of FTIR data, the spectra are consistent with those of previous research [29]. The maintenance of P = O peak which is indicative for the nucleic acids of the phage suggests that the genetic material of the phage is preserved during the encapsulation process. the shift of spectra towards higher wavenumbers may be attributed to the electrostatic interactions that occur between TPP and chitosan and the presence of a new peak of carbonyl group in phage HK6 encapsulated CS-NPs, indicate the appearance of amide bonds of phage protein which prove that phage HK6 had been encapsulated into CS-NPs [30, 31].

According to size distribution study, it was discovered that the phage HK6 encapsulated CS-NPs had a broader size distribution than the CS-NPs alone. The hydrodynamic diameter of CS-NPs was measured at 180 ± 10 nm, while phage HK6 encapsulated CS-NPs showed a larger average size of 297 ± 18 nm. In agreement with various studies in the literature, the reported nanoparticle size is within a range of 100–400 nm [32]. Furthermore, the results agree with definitions claiming that nanoparticles should be sized in the range of 170–580 nm [33], thus confirming their categorization as nanoparticles. Nevertheless, these dimensions vary from different reports that propose nanoparticles should have a size below 100 nm for medical applications [34]. These differences between the sizes is most probably due to differences in the pH levels of the medium, the preparation techniques, and the basic constituents [35].

Additionally, the analysis of the zeta potential of both CS-NPs and phage HK6 encapsulated CS-NPs gave no considerable variation which, in its turn, points to the stability of the nanoparticles. This indicates that the electrostatic properties, which are essential for their functionality, were not disrupted during the encapsulation process [36, 37].

SEM and TEM imaging show an increase in the average size of phage HK6 encapsulated CS-NPs in comparison to empty CS-NPs. These observations confirmed the results of dynamic particle size measurement indicating that Phage encapsulated into CS-NPs.

Thermal and pH stability studies show that phage HK6 encapsulated CS-NPs tolerate a variety of temperatures; ranging of 25–80 °C, while the phage HK6 is heat sensitive in its free form and was completely inactive at 70 °C and 80 °C.

The encapsulation of phage within (CS-NPs) provides a protective microenvironment and stable matrix around the phage, limiting their exposure to heat [20]. Moreover, the pH expression of the phage HK6 encapsulated CS-NPs was also evaluated and it showed a stable nature at pH 3 to 12 for 1 h. Quite differently, free phage HK6 showed no activities under alkaline condition with pH 11 and 12, highlighting the protective effects of the CS-NP encapsulation from the degradation of these extreme pH levels [38]. These findings make the encapsulated phage highly potent to be used in clinical settings compared to the phage only where it can be mixed with alkaline disinfectants, which are frequently employed for the management of various clinical infections [18, 26]. Chitosan has reported shown activity against bacteriophages through its interaction with phage tail fiber or envelope, rendering phage inactive and unable to interact with its bacterial host [39, 40]. For instance, chitosan has showed > 6 log-units’ reductions in bacteriophage phi6 count that was attributed to deformity of phage envelope [39]. Notwithstanding, this was not observed in our example, where encapsulated Enterobacter phage HK6 showed a comparable infectivity and titer to the free form across the pH range of 3–10 and temperature range 25–60 ˚C (Fig. 4). Moreover, it displayed higher stability when tested at extreme condition, pH of 11–12 and temperature ≥ 70 ˚C. The reasonable explanation for this difference may be attributed to the different preparation protocols for the used chitosan. For example, in our study we have used nano formulated chitosan instead of normal one used in other studies.

Controlled release mechanisms modulate the kinetics of phage infection, leading to a delay in the release of active phage and thereby extending the duration during which host bacteria undergo lysis. This prolonged efficacy of phage encapsulated CS-NPs contrasts with the behavior of free phages, which typically demonstrate a diminishing capacity to eradicate host bacteria over time, often accompanied by an increase in phage titers. The protracted delivery and lysis activity associated with controlled release systems hold promise in mitigating the emergence of phage resistance [32].

CS NPs form tightly interconnected network structures, resulting in reduced release rates. Furthermore, the polycationic nature of chitosan chains facilitates electrostatic interactions with the phage, creating an additional barrier that impedes phage diffusion out of the beads.

The release pattern was likely caused by the swelling of the chitosan matrix, which facilitated the phage diffusion and disintegration from the nano-polymer matrix. The results presented here are consistent with the research conducted by Kaikabo [26].

It is a significant finding that phage HK6 encapsulated in CS-NPs exhibit superior antibacterial activity than phage HK6 or CS-NPs alone. Electrostatic interaction between positively charged chitosan molecules and negatively charged lipopolysaccharides (Gram negative bacteria) and teichoic acids (Gram-positive bacteria) may lead to the blocking of intra/extracellular exchanges or even cell wall disruption and, finally, leakage of cytoplasmic content and may disrupt biofilms. Furthermore, this enhances the efficiency of phage delivery to the target bacteria, ensuring that a higher number of phages reach and infect the bacterial cells. resulting in a synergistic effect between the phage and the CS-NPs [26, 41].

The effect of CS-NPs on cell viability was evaluated using MTT assay, the results provide no significant cytotoxicity suggesting this approach as promising step for subsequent preclinical and clinical validation.

## Conclusion

Phage HK6 encapsulated in CS-NPs significantly enhances its stability, efficacy, and delivery against *E. cloacae*, with a high entrapment efficiency of 97% and improved thermal stability up to 80 °C. This encapsulation also ensures sustained phage release and superior antibacterial activity within 2 h of exposure, confirming the potential of CS-NP encapsulated phage HK6 as a safe and effective alternative in phage therapy.

## Electronic Supplementary Material

Below is the link to the electronic supplementary material.

Supplementary Material 1

## Data Availability

The nucleotide sequence of Enterobacter phage vB_EclM_HK6 has been deposited in the NCBI GenBank database (accession number PP337149).

## Abbreviations

CS-NPs: Chitosan nanoparticles
FT-IR: Fourier-transform infrared spectroscopy
SEM: Scanning Electron Microscopy
TEM: Transmission Electron Microscopy
E. cloacae: Enterobacter cloacae
DLA: Double Layer Assay
PFU: Phage forming unit
EE %: Entrapment efficiency
DLS: Dynamic light scattering
FE-SEM: Field emission scanning electron microscope
CCD: Charge coupled device
BCA: Biciconic Acid Assay
TPP: Sodium tripolyphosphate
DMSO: Dimethyl sulfoxide
