# Supplementary material for: Chitosan nano-formulation enhances stability and bactericidal activity of the lytic phage HK6
Source: BMC Biotechnol. 2025 Jan 6;25:3. doi: 10.1186/s12896-024-00934-6 (PMC11705691; doi:10.1186/s12896-024-00934-6)
Supplement: Supplementary file 1 — Supplementary Material 1 [file 12896_2024_934_MOESM1_ESM.docx]

Figure S1: Cell viability (%) of epithelial cell line assessed by MTT assay by treatment with varying chitosan concentrations (0.01 – 1 mg/mL).
